# Editorial: Pediatric endoscopy and sedation—volume II

**DOI:** 10.3389/fped.2024.1403090

**Published:** 2024-04-03

**Authors:** Ron Shaoul, Jennifer R. Lightdale, Andrew S. Day

**Affiliations:** ^1^ Rambam Medical Center, Faculty of Medicine, Pediatric Gastroenterology and Nutrition Institute, Ruth Children’s Hospital of Haifa, Technion, Haifa, Israel; ^2^ Division of Gastroenterology, Hepatology and Nutrition, Boston Children’s Hospital and Department of Pediatrics, Harvard Medical School, Boston, MA, United States; ^3^ Department of Paediatrics, University of Otago Christchurch, Christchurch, New Zealand

**Keywords:** pediatrics—children, endoscopy, transnasal, bowel preparation, simulation, duodenal web

**Editorial on the Research Topic** Pediatric endoscopy and sedation—volume II

Following the success of an earlier research topic that focused on a number of aspects of endoscopic practice in children (1), this research topic aimed to enable further updates in this important and fast expanding area. The four articles included provide some insight into different areas of interest.

Scarallo et al. provided a narrative review on aspects of endoscopic training. They identify and discussed several important aspects, including the importance of a training curriculum, the relevance of train the trainers’ courses and assessment of proficiency. The authors also discuss the emergence and development of simulation-based tools. Simulation has been increasingly shown to provide a safe and effective environment to enhance the development of expertise. One recent evaluation of simulation showed that trainees increased skill development and confidence in endoscopic hemostasis (2).

Transnasal endoscopy (TNE) has emerged in recent years as a new approach for endoscopic assessment in children (3). This endoscopic method is generally considered to be safe, effective and eliminates the requirements for and risks of sedation or anesthesia. Friedlander et al. have outlined the rationale of this endoscopic approach and highlighted practical aspects to be undertaken in developing a TNE programme.

Ileo-colonoscopy is key component of evaluation of the cause of lower gastrointestinal tract symptoms and for the diagnosis of conditions such as inflammatory bowel disease or colonic polyposis. A critical component of the outcome of ileo-colonoscopy is adequate bowel preparation. Various preparations and bowel prep agents are available, but some appear to be more effective than others (4). Dankner et al. report the outcomes of an automated program to enhance bowel preparation outcomes. The Pediatric Colonoscopy Digital Navigation Program was designed to provide instructions, education and reminders to patients. According to physician reporting of bowel preparation quality the 56 children who were managed with this program has superior bowel outcomes than the 60 children managed historically with standard care.

Incomplete duodenal obstruction due to a congenital diaphragm or web, also referred to as a windsock deformity, is a rare condition that typically presents in early life with recurrent vomiting (commonly bile-stained) (5). Contrast studies can illustrate the presence of the web or diaphragm. Traditional management has been lateral duodenotomy and excision of the obstruction. Sun et al. reported on the outcomes of 13 children with this condition who were diagnosed and treated endoscopically. Successful and safe treatment was achieved with endoscopic duodenotomy and balloon dilatation. These data provide further support for this less invasive approach to the management of this condition.

Together these four reports covered some interesting aspects relevant to the field of endoscopy in children and highlight some particular advances and new approaches. It is anticipated that further progress and optimisation of endoscopic approaches and interventions in children will continue in the coming years.
